# Levantamiento del seno maxilar sin injerto óseo e implante simultáneo: un reporte de caso

**DOI:** 10.21142/2523-2754-1202-2024-202

**Published:** 2024-06-27

**Authors:** Brayam Coronel Zabalbu, Oswaldo Andreé Cáceres, Andrea Vergara-Buenaventura

**Affiliations:** 1 Departamento de Periodoncia, Facultad de Ciencias de la Salud, Universidad Peruana de Ciencias Aplicadas. Lima, Perú. brayamcorone1407@gmail.com , peocacer@upc.edu.pe , peandver@upc.edu.pe Universidad Peruana de Ciencias Aplicadas Departamento de Periodoncia Facultad de Ciencias de la Salud Universidad Peruana de Ciencias Aplicadas Lima Peru brayamcorone1407@gmail.com peocacer@upc.edu.pe peandver@upc.edu.pe

**Keywords:** elevación de seno maxilar, técnica transalveolar, implantes dentales, dental implants, maxillary sinus lift, transalveolar technique

## Abstract

Se realizó un levantamiento de seno maxilar sin injerto óseo en una paciente de sexo femenino de 23 años que requería la colocación de un implante dental en la zona edéntula de la pieza 16. La evaluación clínica y tomográfica permitieron planificar y realizar la elevación de seno maxilar mediante el abordaje transcrestal y sin la necesidad de injerto óseo. El reborde residual de 6 mm permitió la colocación de un implante dental simultáneo. Luego de 6 meses de seguimiento tomográfico, antes de la carga protésica, se consiguió una altura ósea vertical de 8,83 mm, lo que dio como resultado una ganancia ósea vertical de 2,83 mm. Adicionalmente, se observa una adecuada integración del implante dental sin complicaciones posoperatorias. El levantamiento del seno maxilar por vía transcrestal, sin el uso de injerto óseo, se muestra como una técnica viable, segura y efectiva para la colocación simultánea de implantes dentales en pacientes con atrofia ósea en el área del maxilar superior. Esta técnica ofrece ventajas como la reducción del tiempo quirúrgico, menor morbilidad, menor costo y un proceso de cicatrización más rápido, comparado con los métodos convencionales.

## Abstract (en)

A maxillary sinus lift was performed without bone grafting in a 23-year-old female patient who required the placement of a dental implant in the edentulous area of tooth 16. The clinical and tomographic evaluation made it possible to plan and perform the maxillary sinus lift using the transcrestal approach and without the need for bone grafting. The residual ridge of 6mm allowed the placement of a simultaneous dental implant. After 6 months of tomographic follow-up, before prosthetic loading, a vertical bone height of 8.83 mm was achieved, resulting in a vertical bone gain of 2.83 mm. Additionally, an adequate integration of the dental implant was observed without postoperative complications. Transcrestal sinus lift without the use of bone graft is shown to be a viable, safe and effective technique for the simultaneous placement of dental implants in patients with bone atrophy in the maxillary area. This technique offers advantages such as reduced surgical time, less morbidity, lower cost and a faster healing process compared to conventional methods.

## INTRODUCCIÓN

Los implantes dentales son sustitutos radiculares de titanio colocados en los huesos maxilares para reemplazar dientes perdidos a causa de la caries dental o la enfermedad periodontal, y se consideran un tratamiento quirúrgico alentador para recuperar la función masticatoria en pacientes edéntulos parciales ^1^. Sin embargo, la colocación de implantes dentales en el maxilar posterior puede considerarse un desafío clínico, pues comúnmente se observa una falta de calidad y cantidad ósea adecuada, como resultado de la neumatización del seno maxilar o la remodelación ósea después de la extracción de una pieza dentaria ^2^. 

En la actualidad, existen diversos tratamientos para el maxilar posterior atrófico, entre los cuales destaca la elevación del seno maxilar ^3^. Esta técnica ha sido ampliamente utilizada en los últimos 30 años con la finalidad de incrementar el volumen óseo en la zona posterior del maxilar y realizar una colocación de implantes simultánea o diferida, con el fin de restituir la función en la zona ^4^. 

La técnica original fue descrita por Tatum ^5^, quien propuso realizar una ventana en la pared lateral del seno maxilar y colocar biomaterial óseo debajo de la membrana de Schneider. Posteriormente, fue publicada por Boyne y James en 1980, usando como material de relleno hueso autógeno procedente de la cresta iliaca ^6^. Sobre las desventajas de esta técnica se han reportado una alta morbilidad asociada, un amplio periodo de cicatrización para la formación del tejido óseo que oscila entre 6 y 10 meses, y un segundo procedimiento quirúrgico para la colocación de los implantes dentales ^7^. 

En busca de mejorar la experiencia y satisfacción del paciente, se propuso la técnica transcrestal o elevación de seno con osteótomos, conocida también como abordaje transcrestal o elevación interna del seno ^8^. Esta utiliza la misma preparación del implante dental para acceder al suelo del seno maxilar y desplazar la membrana sinusal. Su principal ventaja es la baja morbilidad clínica y una reducción significativa del tiempo y el número de fases quirúrgicas ^9^. Adicionalmente, posibilita colocar implantes más largos, aumentar la densidad ósea y el volumen del hueso esponjoso mediante compresión con osteótomos de diámetros progresivos ^8^. 

A la fecha, se han descrito diferentes modificaciones y propuestas del abordaje transcrestal, como el uso combinado de trefinas, piezoeléctricos y sistemas hidráulicos ^10^, así como la decisión de usar o no biomateriales óseos, que brindan ventajas evidentes en términos de rentabilidad y ahorro de tiempo ^11^. Esta idea ha tenido bastante aceptación debido al conocimiento del potencial osteogénico innato de la membrana de Schneider ^12^. 

Por ello, el objetivo de este reporte de caso fue presentar la técnica de levantamiento de seno maxilar mediante el abordaje transcrestal sin injerto óseo y con la colocación de un implante simultaneo, así como las consideraciones quirúrgicas y los posibles desafíos que puedan surgir. Además, se discutieron las ventajas y limitaciones de esta aproximación terapéutica, lo que proporcionó información valiosa para futuros casos y decisiones clínicas. 

## REPORTE DE CASO

Paciente femenino de 23 años, ASA I, acude al Centro de Salud de la Universidad Peruana de Ciencias Aplicadas (CUS-UPC) para colocarle un implante en la zona edéntula de la pieza 1.6. Durante la evaluación, la paciente refiere que hace unos meses se le fracturó la primera molar superior derecha y, como consecuencia, le realizaron la exodoncia atraumática de dicha pieza dentaria.

**Figura 1:**
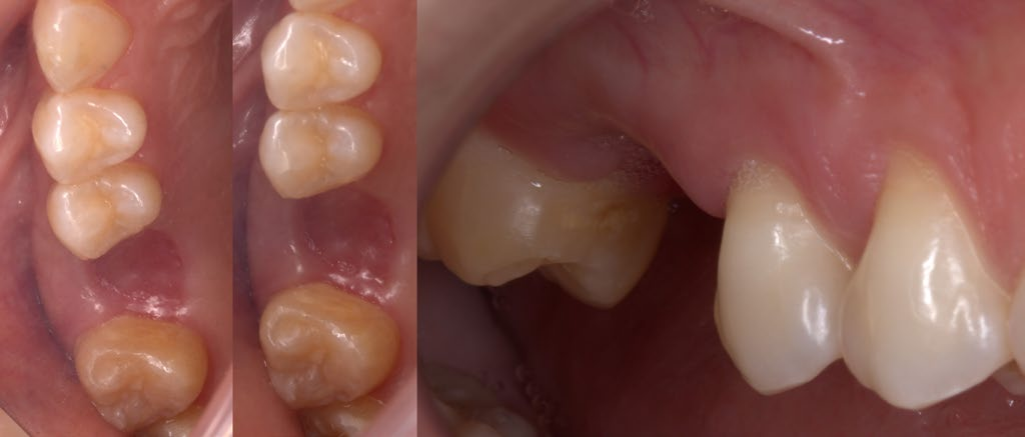
Zona edéntula 16 (vista oclusal y lateral)

**Figura 2:**
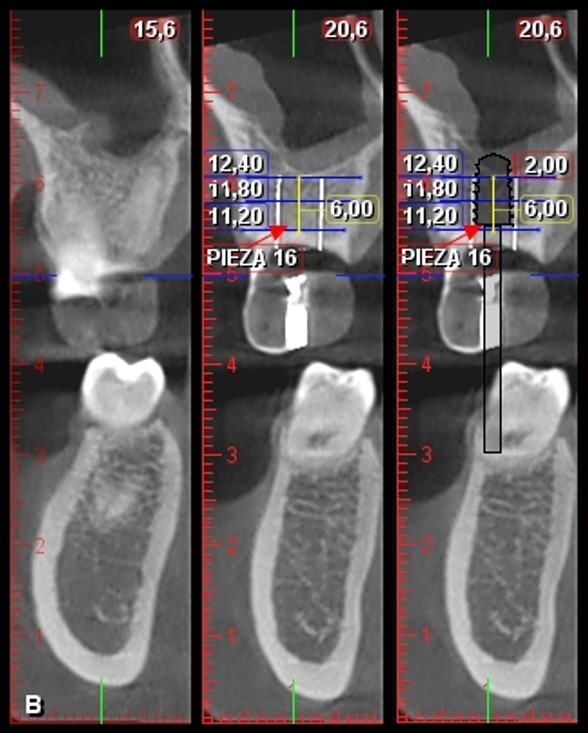
Planificación quirúrgica en tomografía digital

Tras la inspección clínica, se observó la ausencia de la pieza dentaria 1.6, fenotipo gingival delgado y disminución en altura de reborde edéntulo Seibert tipo II ^13^ (Fig. 1). Tomográficamente, se registraron medidas de la altura del reborde óseo residual en relación con el piso del seno maxilar (6 mm) y el ancho de la cresta ósea a nivel cervical (11,20 mm), medio (11,80 mm) y apical (12,40 mm), con lo que se identifica la presencia de un seno maxilar ovoide, según Nie *et al*. ^14^. Se realizó la planificación quirúrgica para la realización de una elevación del seno maxilar con abordaje transcrestal y la colocación de un implante dental de 4,8 x 8 mm (Fig. 2).

**Figura 3:**
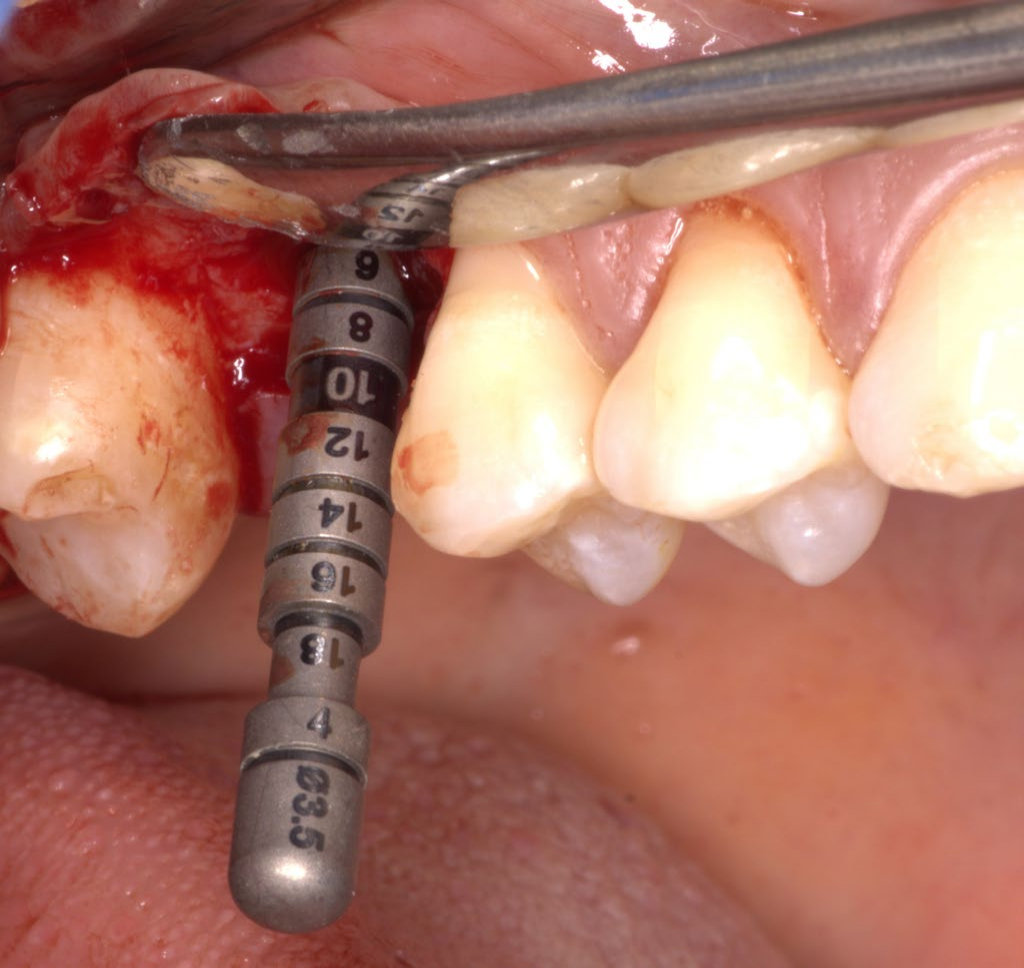
Calibración de profundidad de la secuencia de fresado (6 mm)

**Figura 4:**
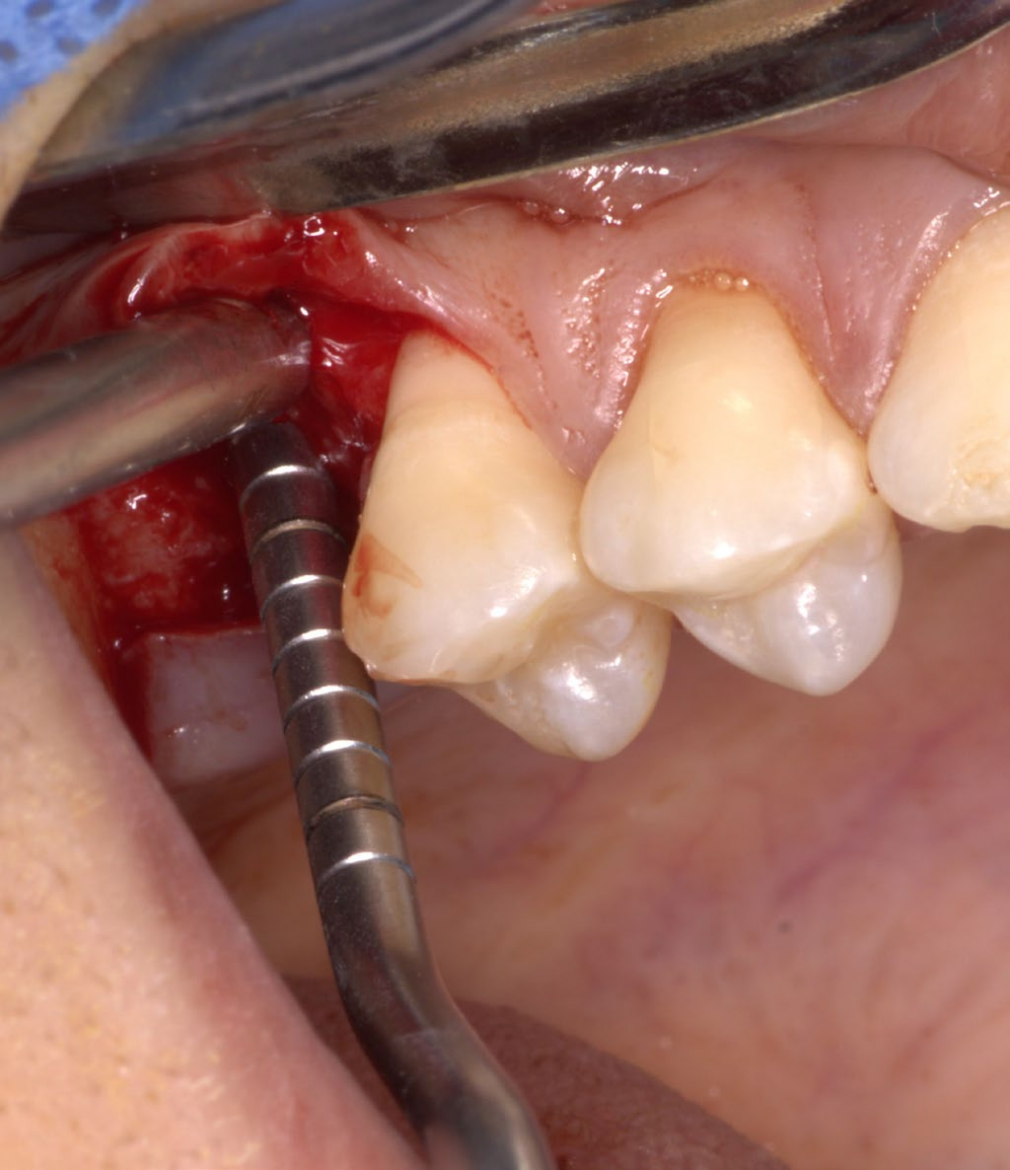
Abordaje transcrestal del seno maxilar (2 mm)

**Figura 5:**
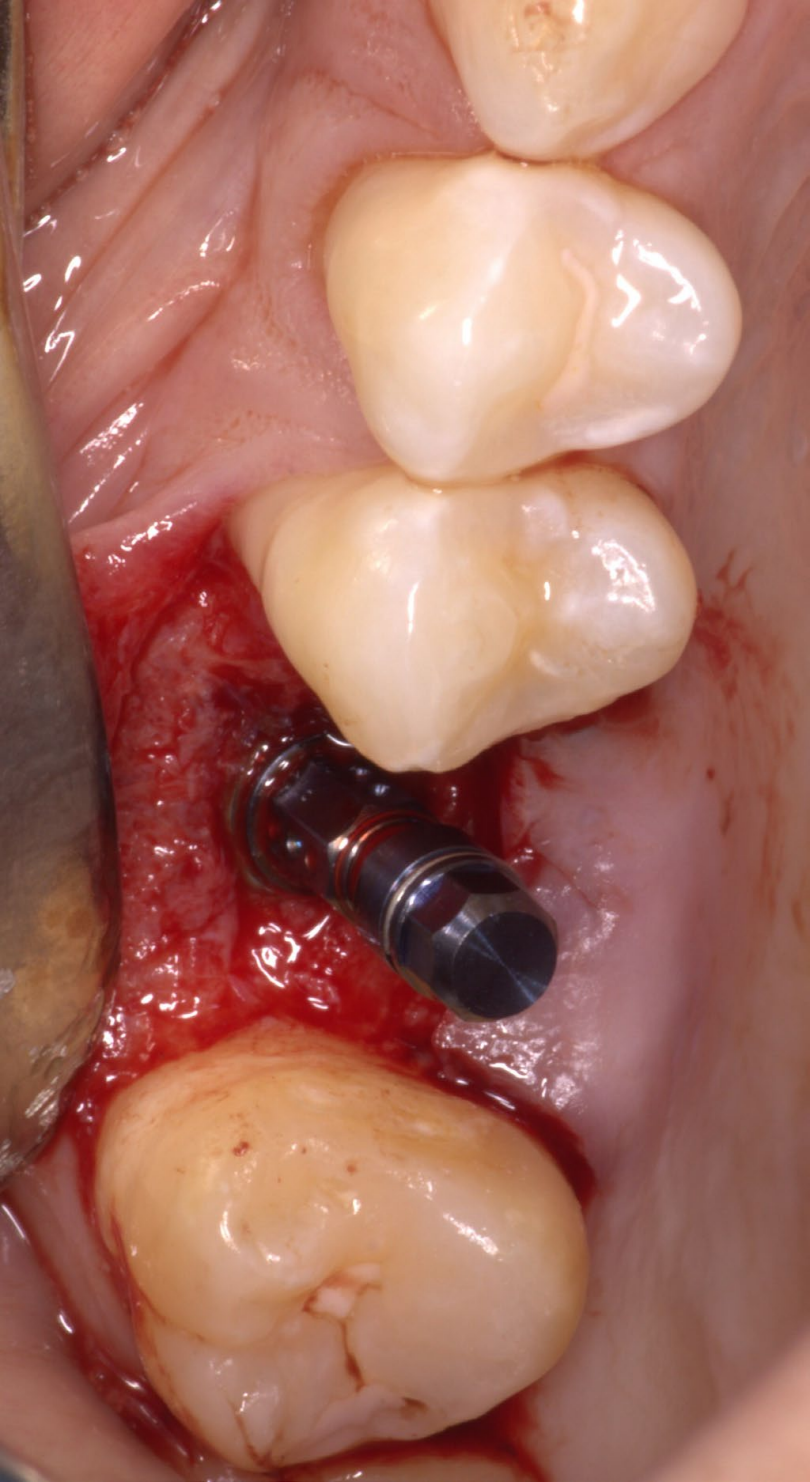
Colocación del implante BLT de φ 4.8 x 8 mm (Straumann)

Se inició el procedimiento quirúrgico, que consistió en lo siguiente:

• Asepsia y antisepsia
• Técnica anestesia infiltrativa por vestibular y palatino de la zona edéntula 1.6 utilizando 2 cartuchos de lidocaína al 2% con epinefrina 1:80.000.
• Incisión supracrestal en la zona edéntula 1.6 e incisiones sulculares en mesial de la pieza dentaria 1.7 y en distal de la pieza dentaria 1.5.
• Decolado del colgajo a espesor parcial y, luego, se procedió a probar la guía quirúrgica para realizar la secuencia de fresado hasta una longitud de 6 mm, dejando 1 mm de distancia al piso del seno maxilar, el cual fue comprobado con los calibradores de profundidad, según el diámetro de la fresa que se utilizó durante la osteotomía (Fig. 3). La secuencia de fresado se realizó hasta la fresa 3,5 mm de diámetro y luego se procedió a realizar el levantamiento de 2 mm del seno maxilar con la técnica de abordaje transcrestal mediante el uso de un osteótomo, hasta llegar a una longitud de 8 mm (Fig. 4).

• Colocación del implante φ 4,8 x 8 mm (Bone Level tapered, Straumann®) y tornillo de cierre, ambos con un torque de inserción de 20 N (Fig. 5).
• Sutura con puntos interrumpidos con ácido poliglicólico 5/0 y lavado de la zona quirúrgica con suero fisiológico al 0,09%.
• Radiografía posquirúrgica del implante en zona edéntula 1.6.
• Indicación de tomar amoxicilina de 500 mg + ácido clavulánico 125 mg cada 8 horas durante 7 días, ketorolaco de 10 mg cada 8 horas por 3 días, cetirizina de 10 mg cada 24 horas por 3 días y clorhexidina al 0,12%, 2 veces al día, por 10 días. Entre las indicaciones posquirúrgicas se le indicó a la paciente reposo absoluto, sin esfuerzo físico, dieta blanda, no sonarse la nariz, no succionar líquidos por dos semanas, no sumergir la cabeza en agua, tampoco estornudar o toser con la boca abierta, mantener la cabeza elevada y aplicarse compresas de hielo durante las primeras 4 horas.

**Figura 6:**
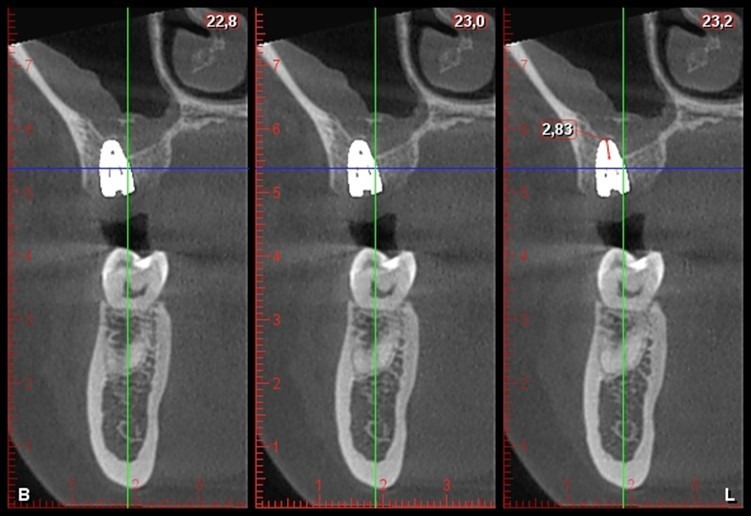
Tomografía posquirúrgica

Se realizó un control posquirúrgico a los 7 días, el cual evidenció tejidos blandos en proceso de cicatrización, por lo que se procedió al retiro de puntos y lavado con suero fisiológico al 0,9%, y se realizó una tomografía de control que evidenció la elevación de seno maxilar, con 2,83 mm aproximadamente (Fig. 6). Asimismo, se realizaron controles posquirúrgicos a los 35 y 56 días, en los cueles se hallaron tejidos blandos en completo proceso de cicatrización.

## DISCUSIÓN

El tratamiento rehabilitador del maxilar posterior puede considerarse un desafío cuando se produce una marcada atrofia ósea por la neumatización del seno maxilar o la remodelación ósea luego de la extracción de una pieza dentaria ^15^. Aunque una alternativa de tratamiento menos invasiva es la colocación de implantes cortos, existen casos en los que la elevación del seno maxilar es necesaria para crear suficiente altura y anchura ósea en el maxilar posterior, y así facilitar la colocación adecuada de implantes dentales ^16^. 

El levantamiento del piso del seno maxilar puede ser realizado utilizando el abordaje mediante una ventana lateral o por el abordaje transcrestal ^17^^,^^18^. La selección de la técnica quirúrgica se basa principalmente en la altura del hueso residual. Cuando se cuenta con más de 5 mm, suele estar indicado el abordaje transcrestal; de lo contrario, será conveniente el abordaje por ventana lateral ^19^. Como principal diferencia entre ambos abordajes, la técnica transcrestal permite elevar el piso del seno maxilar desde un acceso crestal utilizando osteótomos, por lo que resulta mínimamente invasiva y rápida ^8^. Además, permite la colocación inmediata de implantes con un éxito/supervivencia clínica similar a la de los implantes dentales colocados de forma convencional ^20^. 

El presente caso describe la colocación de un implante dental mediante la técnica transcrestal con el uso de osteótomos en una paciente con una altura ósea residual intermedia. La técnica transcrestal permite aumentar la densidad y el volumen del hueso esponjoso en sentido ápico-coronal y bucolingual, por medio de la compresión ^8^, y es considerada superior a la colocación de implantes con ventana lateral en pacientes con rebordes residuales de 4 a 8 mm ^21^. Incluso, se ha descrito que los cambios óseos marginales y las complicaciones posoperatorias son equivalentes al comparar la técnica transcrestal y la colocación de implantes cortos ^21^. La presencia de un reborde residual de 6 mm nos permitió utilizar esta técnica y ganar una altura ósea de 2,8 mm para colocar un implante simultáneo de diámetro regular y de 8 mm de largo. Esta medida concuerda con una revisión previa que reportó una ganancia ósea de 3 a 3,5 mm de altura con el uso de esta técnica ^22^. 

El uso adyuvante de un sustituto óseo es aún tema de debate debido a las pruebas contrastadas sobre su eficacia adicional en los resultados reconstructivos ^23^. Aunque algunos autores recomiendan el uso de injerto óseo para el desplazamiento apical de la membrana sinusal o para tener una mayor ganancia ósea y permitir una dimensión adecuada para la colocación de implantes (24), por motivos económicos, se planificó no colocar ningún tipo de injerto o biomaterial. Esta decisión está fundamentada en el potencial osteogénico innato de la membrana de Schneider demostrado en estudios previos ^12^. Lundgren *et al*. ^25^ demostraron que existe un gran potencial de cicatrización y formación ósea en el seno maxilar sin necesidad de utilizar injertos o sustitutos óseos. La cirugía de levantamiento del seno maxilar permite formar un compartimiento luego de levantar la membrana sinusal, que se llena con sangre y sirve como matriz para la regeneración ósea ^26^. Al comparar histológicamente la estabilidad del implante con el contacto hueso-implante y el área ósea dentro y fuera de las roscas del implante en primates, se encontró que no existen diferencias en la elevación de la membrana sinusal con o sin injerto óseo ^27^. Además, se encontró, en los lugares en contacto con la membrana de Schneider, donde solo existe coágulo, que se deposita hueso nuevo, lo cual indica el potencial osteoinductor de la membrana. 

Del mismo modo, Nedir *et al*. ^28^ han indicado que la presencia del biomaterial no es fundamental para la nueva formación de hueso y la estabilidad del implante incluso con alturas óseas residuales de 3,8 mm ± 1,2 mm. Diferentes estudios han demostrado resultados comparables de la técnica con y sin el uso de injerto óseo ^21^. Taschieri *et al.*^18^ encontraron una tasa de éxito del 98% al año y del 96% a los 5 años en implantes colocados con la técnica transcrestal sin utilizar injertos óseos. 

El levantamiento de seno maxilar mediante la técnica transcrestal ha demostrado ser un procedimiento seguro, eficaz y con una baja incidencia de complicaciones. El presente caso no mostró complicaciones posoperatorias, como la perforación de la membrana de Schneider, dolor, inflamación o hemorragia, que sí se reportaron en otros estudios ^29^. Este enfoque mínimamente invasivo permite reducir el tiempo quirúrgico, la morbilidad posoperatoria, el tiempo de recuperación y el costo, así como brinda mayor satisfacción al paciente ^11^^,^^30^. 

Con las limitaciones de un reporte caso, se puede concluir que el levantamiento del seno maxilar utilizando el abordaje transcrestal con protocolos de colocación simultánea de implantes dentales es un procedimiento eficaz y predecible, que tiene altas tasas de éxito a corto y mediano plazo. Disminuye la morbilidad asociada con la técnica de ventana lateral y, al no usar materiales de injerto óseo, es menos costoso, lleva menos tiempo y no requiere cirugías adicionales en caso se necesite utilizar hueso autógeno. Sin embargo, la decisión final del abordaje dependerá del paciente, el reborde óseo residual, la historia clínica y la condición previa y actual del seno maxilar. Como en todos los abordajes quirúrgicos, la mínima invasión, la simplicidad del procedimiento y la reducción de la morbilidad, es decir, menos complicaciones intraoperatorias y posoperatorias previstas, deben ser de suma importancia para la elección del tratamiento.

## CONCLUSIÓN

El levantamiento del seno maxilar por vía transcrestal sin el uso de injerto óseo en este reporte de caso con atrofia ósea en el área del maxilar superior mostró ser una técnica viable, segura y efectiva para la colocación simultánea del implante dental. Esta técnica ofrece ventajas como reducción del tiempo quirúrgico, menor morbilidad, menor costo y cicatrización más rápida, en comparación con los métodos convencionales, por lo que los clínicos pueden considerarla una alternativa terapéutica eficaz.
